# Regrowth dynamics and morpho-physiological characteristics of *Plantago lanceolata* under different defoliation frequencies and intensities

**DOI:** 10.1371/journal.pone.0310009

**Published:** 2024-09-06

**Authors:** Verónica M. Merino, René Aguilar, Luis F. Piña, Miguel Garriga, Enrique Ostria-Gallardo, M. Dolores López, Felipe Noriega, Jorge Campos, Soledad Navarrete, M. Jordana Rivero

**Affiliations:** 1 Departamento de Producción Animal, Facultad de Agronomía, Universidad de Concepción, Concepción, Chile; 2 Facultad de Agronomía, Universidad de Concepción, Concepción, Chile; 3 Departamento de Producción Animal, Facultad de Ciencias Agronómicas, Universidad de Chile, Santiago, Chile; 4 Departamento de Producción Vegetal, Facultad de Agronomía, Universidad de Concepción, Chillán, Chile; 5 Laboratorio de Fisiología Vegetal, Departamento de Botánica, Facultad de Ciencias Naturales y Oceanográfica, Concepción, Chile; 6 Net Zero and Resilient Farming, Rothamsted Research, North Wyke, Okehampton, Devon, United Kingdom; Institute for Biological Research, University of Belgrade, SERBIA

## Abstract

Traditional pastures in temperate regions face limitations such as reduced growth and nutritional quality during the summer season. Plantain (*P*. *lanceolata* L.) offers advantages like increased yield and decreased nitrogen losses from grazing ruminants. Effective grazing management is essential for pasture health, and defoliation frequency and intensity play a pivotal role. This study aimed to evaluate plantain’s regrowth, yield, and morpho-physiological and chemical responses under different defoliation frequencies and intensities, with the goal of enhancing its management in pastures. The study was conducted in pots within a controlled-environment growth chamber, examining the impact of three defoliation frequencies (based on extended leaf length: 15, 25 and 35 cm) and two defoliation intensities (5 and 8 cm of residual heights) with four replicates (24 pots as experimental units). The variables of interest were morphological characteristics, dry matter (DM) accumulation, herbage chemical composition, growth rate traits, and photosynthetic parameters. Defoliation frequency affected plantain’s growth and nutritional composition. More frequent cuts (15 cm) resulted in lower DM yield per cut and lower stem content, while less frequent cuts (35 cm) produced higher values. Defoliation intensity influenced the proportion of leaves and stems in the total DM, with 5 cm cuts favoring leaves. Nutrient content was also affected by defoliation frequency, with less frequent cuts (35 cm) showing lower crude protein concentration and metabolizable energy content but higher neutral detergent fiber and water-soluble carbohydrate concentration. Plantain’s growth rate variables were mainly influenced by defoliation frequency, with less frequent cuts promoting faster leaf appearance and growth of new leaves. The basal fluorescence variables and chlorophyll content were affected by cutting frequency, being highest when cut less frequently (35 cm), while no differences were found in the actual quantum efficiency among different defoliation frequencies and intensities. The fraction of light dedicated to non-photochemical quenching was highest when cut less frequently and more intensively. Overall, defoliation at 25 cm of extended leaf length balanced plantain forage quality and regrowth capacity.

## Introduction

Pastures are a crucial component of terrestrial ecosystems for feeding grazing ruminants, with a mixture of perennial ryegrass (PRG) (*Lolium perenne* L.) and white clover (WC) (*Trifolium repens* L.) as the dominant pasture type used in temperate regions of the world [1]. These traditional pastures have limited growth and poor nutritional quality over the summer due to soil water stress and plant maturity, reducing herbage dry matter (DM) intake and animal performance [2]. Moreover, the metabolizable energy (ME) content is low (ranging from 8.9 to 10.6 MJ kg^-1^ DM of ME) while the crude protein (CP) content is high (20–25%) throughout most of the year (except in late spring and summer) [3], resulting in an excess of CP intake relative to the nutritional requirements of grazing animals and, subsequently, more nitrogen (N) excreted into the environment, predominantly in urine [4].

Plantain (*Plantago lanceolata* L.) is a perennial herb that has been widely studied for its chemical composition with medicinal properties [5], and for its physiological and ecological characteristics [6]. In the last three decades, plantain has been increasingly used as an alternative forage species in pure and mixed swards to improve DM production and animal performance [7]. Plantain has rapid establishment, similar to PRG [8], and is adapted to a wide variety of soils in temperate regions [9]. It is also more tolerant to water deficit compared to PRG-WC pastures due to its taproots that can access water deeper in the soil profile [10], contributing to maintaining high-nutritive value herbage for ruminants during warm and dry conditions [11]. Mixed pastures containing plantain have shown higher annual herbage DM production than PRG-WC pastures in dryland pastoral systems (17.6 vs. 14.3 t DM ha^-1^ year^-1^, respectively) [12, 13], while pure plantain pastures can produce up to 19.1 t DM ha^-1^ year^-1^ [14]. Relative to PRG pastures, plantain-based pastures (PP, including pure and mixed pastures) have similar CP content (averaging 19.7% for PRG and PP). However, plantain provides higher nutritive value for grazing ruminants due to its lower structural carbohydrates (43.4 vs. 32.1% DM of neutral detergent fiber [NDF] for PRG and PP, respectively) and greater water-soluble carbohydrate (WSC) content (21.1 vs. 29.9% DM for PRG and PP, respectively) [15]. Additionally, plantain offers 1.5 times more macrominerals [16] than PRG. Dairy cows fed plantain-containing pasture reduced total urinary nitrogen (UN) excretion by 22% and UN concentration (a key determinant of N leaching risk) [17] by 30% compared to dairy cows fed PRG [13]. This reduction in N losses increased daily milk production and milk protein yield by 1.02 kg cow^-1^ and 23.4 g cow^-1^, respectively [13]. In addition, cows fed a diet including plantain produce 15–28% less methane (CH_4_) per unit of DM intake (DMI) compared to PRG-based pastures [18]. It has been suggested that to achieve a substantial reduction in UN-loading onto paddocks, the proportion of plantain in pastures grazed by cows should be at least 30% of the DM of the botanical composition [19, 20]. However, some studies have indicated that maintaining the proportion of plantain in sown pastures for an extended period, i.e., more than three years after sowing, could be difficult to achieve at the farm level due to poor pasture persistence [21, 22]. This limited persistence in intensively grazed ruminant production systems has been attributed to its low carbohydrate storage capacity, especially under severe grazing management or strong plant competition, particularly in grass-based pastures [22]. Therefore, the use of PP will likely be limited until there is more certainty about the adequate grazing management that can extend plantain persistence and achieve and maintain this target content in mixed permanent pastures.

Grazing management is considered the most important factor in the regrowth capacity, herbage DM production, persistence [23] and nutritional characteristics of permanent pastures [24]. Grazing affects the herbage’s capacity to regrow through a direct effect on individual plants. This effect involves removing part of their photosynthetically active biomass and carbon reserves mainly stored as WSCs [25, 26]. Grazing also has an indirect effect by opening the canopy and subsequently reducing aboveground competition for light and soil nutrients [27]. The plant’s capacity to restore leaf area and resume photosynthetic activity after grazing depends on defoliation frequency (the time between two consecutive defoliation events) and/or defoliation intensity. The latter is measured as the amount of herbage mass removed in each defoliation event and, therefore, residual height [28].

Herbage DM production and persistence of plantain pasture are mainly affected by the time to the first grazing [14] and defoliation frequency and, to a lesser extent, by defoliation intensity, since most of the WSC reserves are stored in the roots [24]. Plantain pastures must be first defoliated with at least six fully developed leaves to grow sufficient roots for plant survival [14]. Concerning the defoliation frequency of plantain pastures, most studies have defined defoliation based on the number of days between grazing events [29–32]. Ayala et al. [33] found that the herbage DM production of a 3-year-old plantain pasture under sheep grazing was higher when grazed every three weeks compared to every six weeks. The 3-week grazing frequency led to a 67.0% increase in herbage DM production in spring and a 73.3% increase in summer. Likewise, the plantain contribution to total DM in summer increased by 62.6% when a more frequent grazing interval was used. Navarrete et al. [32] found that the proportion of plantain in pure pastures grazed by lactating dairy cows to an 8-cm residual was between 2.7 and 46.3% higher with a grazing frequency of four weeks compared to two weeks.

Defoliation frequency based on rotation days has several limitations when used to indicate when to graze. It does not account the changes in plant morphology or regrowth stage between years or throughout the year, which can reduce plant survival and herbage yields [26]. Furthermore, measuring defoliation frequency in days is not an adequate animal-related indicator; it applies only to situations like those in which the studies were conducted. Defoliation frequency based on leaf regrowth stage has been considered a more sensitive indicator for grazing management than days of rotation. This method considers leaf maturity, which influences photosynthetic capacity, herbage quality for ruminant nutrition, and the status of WSC reserves in roots and stubble. These reserves are known to be used by the plant to initiate and sustain growth when the plant’s photosynthetic capacity is limited, thus maximizing herbage DM production and persistence [26].

Lee et al. [24] evaluated the effects of defoliation frequency of pure plantain pastures (‘Ceres Tonic’ cultivar originating from northern Portugal and selected for its highly erect growth habit, large leaves, and winter activity) [34] based on the extended leaf length (ELL) of 15, 25, 35 and 45 cm, and the defoliation intensity, by cutting at 3–5 cm and 6–8 cm, on DM yield, nutritive characteristics, and plant density over an 18-month period. The study found that pastures defoliated at 45 cm, which represents the lowest frequency of defoliation, increased the total DM yield by up to 39%. However, this increase came at the expense of a decrease in herbage nutritive value. Thus, Lee et al. [24] recommended defoliating plantain pastures at 25 cm of ELL to balance between herbage DM production and nutritive value.

Despite the positive impacts on the environment and productivity of livestock systems, there is limited information on how defoliation frequency (as measured by the ELL) affects the morpho-physiological responses of commercial plantain cultivars, such as compensatory growth and changes in photosynthetic capacity. As a result, the use of PP is likely to remain limited until more is known about the effective management strategies for maintaining the proportion of plantain in pastures over time. This study aimed to evaluate the regrowth capacity, DM yield, and morpho-physiological characteristics of plantain under different defoliation frequencies (based on the ELL) and intensities (residual heights).

## Materials and methods

The study was conducted in a 19 m^2^ growth chamber at the Laboratory of Forage Species, Universidad de Concepción, Chile, from December 2021 to September 2022. The environmental conditions were controlled to maintain maximum and minimum temperatures (daily ranges) of 26°C and 16°C, respectively, with a 14/10-hour light/dark photoperiod and relative humidity ranging from 45% to 70%. Air temperature and humidity inside the growth chamber were recorded daily at one-hour intervals using a thermochron datalogger (iButtons DS1923, Maxim Integrated Products Inc., USA). Light conditions were regulated using two 1000-watt LED systems (30 cm × 50 cm, ECO LED-1000, ProGarden, China) with a photosynthetic photon flux density of 150–170 μmol m^-2^ s^-1^. The light spectrum consisted of 68% 660 nm, 12% 460 nm, 14% white light, 4% 730 nm, and 2% 410 nm. The distance between the light panels and the pots was adjusted until the photosynthetic active radiation (PAR) at the plant level reached 400 μmol m^-2^ s^-1^, which is considered a moderate PAR condition [35]. Additionally, a PAR of 100 μmol m^-2^ s^-1^ was measured at ground level [36]. The PAR was determined using a quantum sensor (SQ-521, Apogee, United States).

### Treatments and experimental design

The experiment followed a completely randomized design with a factorial arrangement involving three defoliation frequencies (ELL of 15, 25 and 35 cm) and two defoliation intensities (5 and 8 cm of residual heights), with four replicates (24 pots in total as experimental units). Defoliation frequency was based on the time required for leaves to grow to the targeted ELL.

### Plant establishment and management

Plantain plants were established in 11 L square plastic pots (25 cm width × 25 cm length × 27 cm height). ‘Ceres Tonic’, a cultivar originating from northern Portugal selected for this study due to its highly erect growth habit, large leaves and winter activity, was used due to its widespread use in temperate climate grazing systems, such as those in southern Chile, and its availability in the local Chilean market. Seeds were pre-germinated in a 10 cm diameter petri dish on December 2, 2021, and transplanted into a 50-cell plant nursery with a height of 10 cm a week later when radicles reached a length of 0.5 to 2 cm. After three weeks, four seedlings were retransplanted and evenly distributed to each pot filled with 1.5 kg of dry soil consisting of a mixture of 80% peat (Kekkila Professional), 10% perlite, and 10% vermiculite. Before the start of the experiment on February 8, 2022, all pots were fully irrigated and their saturated weights were recorded when all excess water had drained away and the pots reached a constant weight, following the methodology of Earl [37].

The defoliation treatments were applied when all plants had reached at least six fully developed leaves (15 March 2022) as recommended by Powell et al. [14]. Pots were matched for the number of mature leaves per plant and ELL per plant, averaging 13.5 mature leaves per plant and 22.4 cm per plant, and were randomly assigned to the six defoliation treatment groups. Plants were irrigated weekly and fertilized with 2.8 g L^-1^ Phostrogen soluble fertilizer (13% N, 10% P, 27% K) to ensure adequate nutrient availability. Soil moisture content in all pots was maintained between 75% to 100% of their field capacity (water-holding capacity). This was achieved by weighing the pots every week using a 30 kg capacity, 0.5 g precision scale (DY208, Maigas, China) and adding the required amount of water to maintain pot weights constant at the target field capacity (an average volume of 400 mL per pot). These management practices were maintained throughout the experimental period, with defoliation occurring 6 to 20 times over the experiment depending on the treatment, with an average of 8.6 to 27.5 days between defoliations (Table 1). To avoid incident light and temperature differences between treatments, pots were randomly moved every week. Temperature data (daily maximum and daily minimum) were used to calculate the accumulation of temperature growing degree-days (GDD) during plantain regrowth using 5°C as the base temperature [24] (Table 2).

**Table 1:** Number of defoliations, accumulated growing degree-days (AGDD) and the average number of days between two consecutive defoliation events at three frequencies (ELL, 15, 25 and 35 cm extended leaf length) and two intensities (5 and 8 cm).

	5 cm of residual height			8 cm of residual height		
**ELL**	**15**	**25**	**35**	**15**	**25**	**35**
**Number of defoliation events**	17	10	6	20	12	6
**AGDD**	156	247	478	132	222	458
**Days between defoliation events**	10	16	24	9	14	28

**Table 2:** Length of extended leaves and dry matter (DM) weight of leaves and stems of plantain (*Plantago lanceolata* L.) plants cut at three frequencies (15, 25 and 35 cm extended leaf length) and at two intensities (5 and 8 cm). abc: Different letters within a row under the frequency columns indicate differences in mean values (least significant difference (LSD), p ≤ 0.05). SEM: Standard error of the mean. Degrees of freedom of ‘Frequency’ effect: 2; Degrees of freedom of ‘Intensity’ effect: 1; Degrees of freedom of the ‘Interaction effect’ (F × I): 2; Degrees of freedom of the Residual: 18.

	Frequency (F)			Intensity (I)			F statistics and p values		
Variable	15	25	35	5	8	SEM	Frequency (F)	Intensity (I)	F × I
**Length of extended leaves (cm)**	15.2^c^	25.4^b^	35.1^a^	25.2	25.3	0.34	**F = 1,704** **p < 0.001**	F = 0.11 p = 0.744	F = 0.22 p = 0.809
**Total DM weight per cut per pot (g)**	0.47^c^	1.34^b^	5.06^a^	2.21	2.36	0.280	**F = 152.2** **p = < 0.001**	F = 0.44 p = 0.518	F = 0.78 p = 0.473
**Leaf DM weight as % total DM**	99.3^a^	96.4^b^	82.7^c^	94.5	91.1	1.191	**F = 110.6** **p = < 0.001**	**F = 11.7** **p = 0.003**	F = 1.84 p = 0.188
**Stem DM weight as % total DM**	0.70^c^	3.65^b^	17.3^a^	5.55	8.88	1.191	**F = 110.6** **p = < 0.001**	**F = 11.7** **p = 0.003**	F = 1.84 p = 0.188
**DM mass accumulated per period (g/pot)**	9.42^c^	16.0^b^	30.4^a^	17.3	19.8	1.917	**F = 62.4** **p < 0.001**	F = 2.51 p = 0.131	F = 0.37 p = 0.695
**DM mass accumulated per period (g/m** ^**2**^ **)**	235^c^	399^b^	759^a^	433	495	47.9	**F = 62.4** **p < 0.001**	F = 2.51 p = 0.131	F = 0.37 p = 0.695

### Measurements

Every three days, the ELL was measured using a randomly selected fully expanded leaf per plant (16 leaves per treatment, 96 leaves in total) with a graduated ruler (measured from the ground to the upright leaf tip) as described by Lee et al. [24]. Once the average ELL reached the target height (15, 25, 35 cm) for each treatment, the herbage of each pot was harvested by cutting it to a residual height of either 5 or 8 cm using hand-shears. During each cutting, the fresh weight of the harvested herbage was recorded using a 0.1-mg precision analytical scale (M214Ai, BEL Engineering, Italy). The leaves, stems and dead material were sorted for morphological composition analysis, then dried in a 125 L forced air oven (BOV V-125F, BioBase, China) at 60°C until a constant weight was achieved and weighed to determine their contribution to the total DM.

Composite samples of approximately 100 g each (composed of all dry herbage cuttings from each pot at its specific residual heights, with 4 samples per treatment, 24 in total) were ground, sieved through a 1 mm mesh and stored for chemical analysis.

The methods of the Association of Official Analytical Chemists [38] were used to estimate DM (method 2001.12), CP (method 2001.11) and ash concentrations (method 942.05). The NDF was estimated according to the method of Van Soest [39]. Metabolizable energy was calculated using the following equations: ME (Mcal kg^-1^ DM) = (1.01 × digestible energy (DE))– 0.45 [40], with the value of DE estimated using the equation DE (Mcal/kg DM) = 5.151 –(0.0535 × NDF) [41].

During three regrowth periods in May, June, and August 2022, one plant per pot (4 plants per treatment, 24 plants in total) was randomly selected and marked with a colored clip to evaluate the regrowth rate of plantain plants, including daily leaf growth rate and leaf and stem emergence rate. Every 3 days, the presence of new leaves and stems was observed on the marked plants to determine the rate of leaf emergence (measured in days per leaf and days per stem). A new leaf or stem was considered “emerged” when its tip became visible [42]. The length of each new leaf emerging on each marked plant and one immature residual leaf per pot was measured during the regrowth period from tip to base using a graduated ruler to measure the elongation rate (cm day^-1^). The number of mature fully expanded leaves on each marked plant was also recorded.

At the end of the study (September 2022), a non-destructive evaluation was conducted to compare the treatment effects on physiological and morphological traits of plantain. Physiological leaf traits, including measurements in light-adapted recently fully extended leaves, such as minimum chlorophyll fluorescence (Fo´), maximum chlorophyll fluorescence (Fm´), steady‐state chlorophyll fluorescence (Fs), linear electron flow (LEF), actual quantum efficiency of PSII (Fv′/Fm′), total non-photochemical quenching (NPQt), quantum yield of PSII (Φ2), fraction of light dedicated to non-photochemical quenching (ΦNPQ), the fraction of light lost due to non-regulated photosynthesis inhibitor processes (ΦNO), fraction of PSII open centres (qL), and relative chlorophyll content (SPAD), were evaluated using a MultispeQ v2.0 device (PhotosynQ Inc., MI, USA) linked to the PhotosynQ [43]. The manufacturer-designed Photosynthesis RIDES 2.0 protocol (photosynQ.org) was employed. Measurements were conducted on three young and fully developed leaves per plant, with one measurement per leaf in the middle third of each leaf. The number of mature fully developed leaves per plant and the number of plants per pot were recorded. Fresh biomass was harvested using hand-shears and then separated into shoots, live leaves, live stems, roots, and dead material (dead leaves and stems). Samples were oven-dried at 60°C and weighed using an analytical scale to determine their contribution to the total DM.

### Statistical analyses

#### Univariate analysis

Except for the chemical characterization, the individual measurements recorded in each regrowth cycle for each experimental unit (pot) were averaged across all the harvests performed in each treatment. Thus, each experimental unit had one average value for each variable. The chemical analysis was performed on one composite sample per experimental unit, pooled across the regrowth cycles. The one-off non-destructive measurement performed in three leaves per experimental unit at the end of the study to assess the physiological and morphological traits was averaged to obtain one value for each variable for each experimental unit. The data were analyzed using a two-way analysis of variance (ANOVA), with the factors defoliation frequency (three levels) and defoliation intensity (two levels) with four replicates. Fisher’s least significant difference (LSD) was used for the statistical separation of means when the ANOVA results were significant (p-value <0.05). All analyses were performed using Genstat 22 (©VSN International Ltd., Hemel Hempstead, UK).

#### Multivariate analysis

To elucidate the relationships among the growth variables and treatments, a Principal Component Analysis (PCA) was performed. The PCA was conducted using all the measured variables for morphological and physiological traits, the growth rate of leaves and stems, and nutritional composition. While the PCA was calculated using all those variables, due to the large number of variables, loadings are only presented for those variables with loadings higher than 0.20 or lower than -0.20 for the first principal component (PC1) or second principal component (PC2). The frequency, intensity, and the interaction effect of the two were tested for significant associations with PC1 and PC2 using an aligned rank transformation analysis of variance (ART ANOVA). PCA, ART ANOVA and figures were performed in R (i386 4.1.2) and R Studio (2021.09.1) using packages ARTool, ggplot2, ggfortify, and Cairo.

## Results

As intended, the average ELL differed between frequencies (p < 0.001), closely matching the target values (Table 2). All the measured variables varied with frequency (p < 0.001). The most frequent cuts (15 cm) yielded the lowest total DM weight per cut, stem DM weight as a percentage of the total DM, and DM accumulation. In contrast, the least frequent cuts (35 cm) displayed the highest values, with the intermediate frequency (25 cm) showing intermediate values for these variables. Conversely, leaf DM weight as a percentage of the total DM exhibited the opposite trend, i.e., the most frequent cuts (15 cm) resulted in higher relative leaf weights compared to stem weights. Nevertheless, the proportion of leaves in the total DM exceeded 80% in all treatments. Regarding DM percentages measured at each cut, the 35-cm frequency showed higher values than the other two frequencies. The only variable affected by defoliation intensity was the percentage of DM partitioned into leaves (p = 0.003) and stems (p = 0.003). This indicated that leaves constituted a higher proportion of the total DM when cut at a lower height (5 cm), whereas stems did not (Table 2). None of the variables presented an interaction effect (p > 0.05).

Regarding nutritional components, cutting frequency affected all components except for ash content. The DM content of the harvested material was 1 percentage point higher (p < 0.01) when plants were cut at a leaf length of 35 cm, compared to the other two frequencies, on average. Similarly, NDF and WSC exhibited the same pattern, showing a 4.1 percentage points increase (p = 0.044) in the case of NDF and a 1.40 percentage points increase (p = 0.047) in the case of WSC when cut least frequently (35 cm). In contrast, CP content was 3 percentage points lower (p < 0.01) under the least frequent cutting conditions. Cutting intensity only had an impact on ash content (p = 0.005), where plants cut at 5 cm had 0.9 percentage points higher mineral content compared to those subjected to 8 cm cutting intensity. None of the variables exhibited an interaction effect (p > 0.05; Table 3).

**Table 3:** Nutritional composition of plantain (*Plantago lanceolata* L.) plants cut at three frequencies (15, 25 and 35 cm extended leaf length) and at two intensities (5 and 8 cm). abc: Different letters within a row under the frequency columns indicate differences in mean values (least significant difference (LSD), p ≤ 0.05). SEM: Standard error of the mean. Degrees of freedom of ‘Frequency’ effect: 2; Degrees of freedom of ‘Intensity’ effect: 1; Degrees of freedom of the ‘Interaction effect’ (F × I): 2; Degrees of freedom of the Residual: 18.

	Frequency (F)			Intensity (I)			F statistics and p values		
Variable	15	25	35	5	8	SEM	Frequency (F)	Intensity (I)	F × I
**Dry matter (DM) percentage at cutting**	6.98^b^	7.24^b^	8.18^a^	7.43	7.49	0.233	**F = 14.7** **p = < .001**	F = 0.09 p = 0.762	F = 1.17 p = 0.334
**Crude protein, as % DM**	30.7^a^	32.1^a^	28.4^b^	29.7	31.1	0.80	**F = 11.6** **p = < .001**	F = 4.13 p = 0.057	F = 2.30 p = 0.129
**Neutral Detergent Fibre, as % DM**	25.6^b^	25.1^b^	29.5^a^	26.3	27.1	1.78	**F = 3.72** **p = 0.044**	F = 0.33 p = 0.574	F = 0.42 p = 0.664
**Water Soluble Carbohydrates, as % DM**	3.95^b^	4.01^b^	5.38^a^	4.55	4.35	0.599	**F = 3.64** **p = 0.047**	F = 0.17 p = 0.685	F = 0.14 p = 0.873
**Metabolizable Energy, MJ/kg DM**	14.1^a^	14.2^a^	13.2^b^	13.9	13.8	0.403	**F = 3.72** **p = 0.044**	F = 0.33 p = 0.574	F = 0.42 p = 0.664
**Ash, as % DM**	19.3	19.3	18.6	19.5	18.6	0.34	F = 2.69 p = 0.095	**F = 10.1** **p = 0.005**	F = 0.26 p = 0.773

All growth rate variables of plantain, except for days per stem, were affected by defoliation frequency (Table 4). Days per stem had an overall mean (±SEM) of 6.13 ± 1.839 days. The leaf appearance rate was 65% faster for the highest frequency (15 cm) (p = 0.005) compared to the other two frequencies, which did not differ from each other (averaging 4.05 d). Concerning the growth rate of the residual leaves, the two most frequent cutting regimes (15 and 25 cm) exhibited 54% higher values (averaging 1.30 cm/d) compared to the least frequent cuts. On the other hand, the total number of leaves and stems at cutting, as well as the total number of new leaves, were highest (p < 0.001) for the least frequent cuttings (35 cm) and lowest for the most frequent cutting regime, with the intermediate frequency presenting intermediate values for these variables. The only variable affected by cutting intensity was the growth rate of the residual leaves, as leaves had a 9% faster growth rate when cut at a greater height (8 cm) compared to the more intensive cutting regime (Table 4). No variables showed an interaction effect (p > 0.05).

**Table 4:** Growth rate variables of leaves and stems of plantain (*Plantago lanceolata* L.) plants defoliated at three frequencies (15, 25 and 35 cm extended leaf length) and two intensities (5 and 8 cm). abc: Different letters within a row under the frequency columns indicate differences in mean values (least significant difference (LSD), p ≤ 0.05). SEM: Standard error of the mean. Degrees of freedom of ‘Frequency’ effect: 2; Degrees of freedom of ‘Intensity’ effect: 1; Degrees of freedom of the ‘Interaction effect’ (F × I): 2; Degrees of freedom of the Residual: 18.

	Frequency (F)			Intensity (I)			F statistics and p values		
Variable	15	25	35	5	8	SEM	F	I	F × I
**Phyllochron interval (d/leaf appearance)**	6.68^a^	3.95^b^	4.15^b^	5.22	4.62	0.807	**F = 7.08** **p = 0.005**	F = 0.83 p = 0.375	F = 1.11 p = 0.351
**Growth rate of residual leaves (cm/d)**	1.26^a^	1.33^a^	0.84^b^	1.09	1.19	0.044	**F = 73.1** **p = <0.001**	**F = 7.84** **p = 0.012**	F = 1.54 p = 0.241
**Growth rate of new leaves (cm/d)**	0.94^b^	1.22^a^	1.12^a^	1.08	1.11	0.054	**F = 13.4** **p = <0.001**	F = 0.30 p = 0.591	F = 0.69 p = 0.514
**Total number of fully developed leaves**	3.48^c^	6.59^b^	10.92^a^	6.96	7.03	0.491	**F = 116.3** **p = < 0.001**	F = 0.04 p = 0.854	F = 2.98 p = 0.076
**Total number of new leaves**	1.97^c^	4.74^b^	9.21^a^	5.38	5.23	0.468	**F = 121.7** **p = <0.001**	F = 0.15 p = 0.699	F = 2.95 p = 0.078
**Stem appearance rate (d/stem)**	4.81	7.66	5.92	5.27	7.00	1.839	F = 1.22 p = 0.318	F = 1.33 p = 0.263	F = 1.13 p = 0.344
**Total number of stems at cutting**	0.38^c^	2.09^b^	6.95^a^	2.75	3.52	0.498	**F = 94.0** **p = <0.001**	F = 3.64 p = 0.073	F = 0.58 p = 0.568

**Fig 1:**
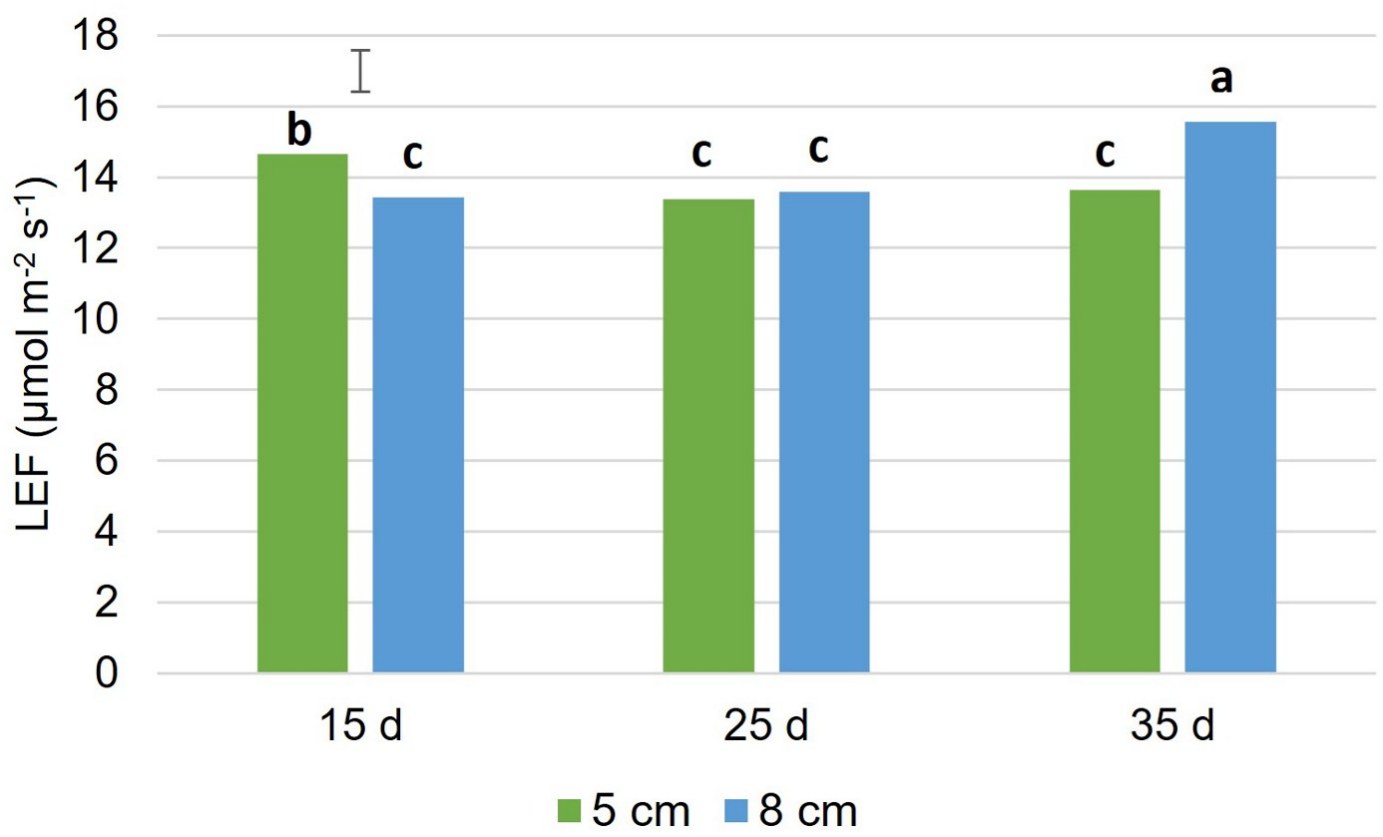
Linear electron flow (LEF, μmol m^-2^ s^-1^) of leaves of plantain (*Plantago lanceolata* L.) plants cut at three frequencies (15, 25 and 35 cm extended leaf length) and at two intensities (5 and 8 cm). Floating bar: standard error of the mean/ Least significant difference (p ≤ 0.05) of the interaction effect was 1.236.

Some of the chlorophyll fluorescence variables did not vary with cutting frequency or intensity (p > 0.05). Specifically, Fv’/Fm’, NPQt and ΦNO had overall means of 0.737 ± 0.0105 units, 0.810 ± 0.1922 units and 0.254 ± 0.0081 units, respectively (Table 5). The qL showed a tendency (p = 0.05) to vary with cutting intensity but not with frequency (p = 0.300), with a trend towards a 9.6% increase in the mean value when cut at 8 cm compared to 5 cm (Table 5). Regarding Φ2, it varied with cutting intensity (p = 0.009), with a mean value 6.4% higher when cut at 8 cm. Additionally, Φ2 tended (p = 0.084) to be greater when cut at 15 cm compared to 35 cm. The basal fluorescence variables were affected by cutting frequency; Fm’ (p < 0.001), Fo’ (p < 0.001), and Fs (p = 0.002) were highest when cut less frequently (35 cm) and lowest when cut more frequently (15 cm). ΦNPQ was affected by both cutting frequency (p = 0.041) and intensity (p = 0.032); ΦNPQ was highest when cut least frequently (35 cm) and when cut more intensively (5 cm) compared to the other levels (Table 5). LEF was the only fluorescence parameter that exhibited an interaction effect (F_2,18_ = 7.20; p = 0.005). Interestingly, the most extreme cutting frequency values (15 cm vs. 35 cm) showed opposite responses to cutting intensity; LEF was higher when cut more intensively (5 cm) under the most frequent cutting regime, while it was lower when cut least frequently, presenting the highest mean value under this combination (Fig 1). Similar to the basal fluorescence variables, chlorophyll content (SPAD index) also increased with lower defoliation frequency (Table 5).

**Table 5:** Chlorophyll fluorescence parameters and chlorophyll content of plantain (*Plantago lanceolata* L.) leaves cut at three frequencies (15, 25 and 35 cm extended leaf length) and two intensities (5 and 8 cm). abc: Different letters within a row under the frequency columns indicate differences in mean values (least significant difference (LSD), p ≤ 0.05). SEM: Standard error of the mean. Degrees of freedom of ‘Frequency’ effect: 2; Degrees of freedom of ‘Intensity’ effect: 1; Degrees of freedom of the ‘Interaction effect’ (F × I): 2; Degrees of freedom of the Residual: 18. Fo´: minimum chlorophyll fluorescence. Fm´: maximum chlorophyll fluorescence. Fs: steady‐state chlorophyll fluorescence. LEF: linear electron flow. Fv′/Fm′: actual quantum efficiency of PSII, NPQt: total non-photochemical quenching. Φ2: quantum yield of PSII. ΦNO: the fraction of light lost due to non-regulated photosynthesis inhibitor processes. ΦNPQ: fraction of light dedicated to non-photochemical quenching. qL: fraction of PSII open centers. SPAD: relative chlorophyll content.

	Frequency (F)			Intensity (I)			F statistics and p values		
Variable	15	25	35	5	8	SEM	F	I	F × I
**Fm´**	4603^c^	4839^b^	5058^a^	4767	4899	93.9	**F = 11.8** **p < 0.001**	F = 2.98 p = 0.101	F = 1.18 p = 0.329
**Fo´**	1156^b^	1245^b^	1392^a^	1266	1262	66.6	**F = 12.7** **p < 0.001**	F = 0.01 p = 0.908	F = 0.83 p = 0.452
**Fs**	1922^b^	2138^ab^	2273^a^	2166	2056	84.8	**F = 8.75** **p = 0.002**	F = 2.50 p = 0.131	F = 0.70 p = 0.511
**Fv´/Fm´**	0.748	0.740	0.723	0.733	0.740	0.0105	F = 3.00 p = 0.075	F = 0.61 p = 0.446	F = 2.89 p = 0.082
**NPQt**	0.650	0.735	1.045	0.812	0.808	0.1922	F = 2.34 p = 0.125	F = 0.00 p = 0.983	F = 1.70 p = 0.210
**Φ2**	0.583	0.554	0.551	0.545	0.580	0.0146	F = 2.85 p = 0.084	**F = 8.68** **p = 0.009**	F = 1.43 p = 0.265
**ΦNO**	0.255	0.259	0.248	0.285	0.250	0.0081	F = 0.99 p = 0.391	F = 1.27 p = 0.274	F = 1.74 p = 0.203
**ΦNPQ**	0.165^b^	0.184^ab^	0.205^a^	0.198	0.171	0.0145	**F = 3.83** **p = 0.041**	**F = 5.42** **p = 0.032**	F = 2.25 p = 0.134
**qL**	0.481	0.448	0.482	0.449	0.492	0.0247	F = 1.29 p = 0.300	**F = 4.43** **p = 0.050**	F = 1.08 p = 0.362
**SPAD**	38.7^c^	45.4^b^	53.4^a^	45.3	46.4	1.62	**F = 41.1** **p < 0.001**	F = 0.80 p = 0.384	F = 0.67 p = 0.525

**Fig 2:**
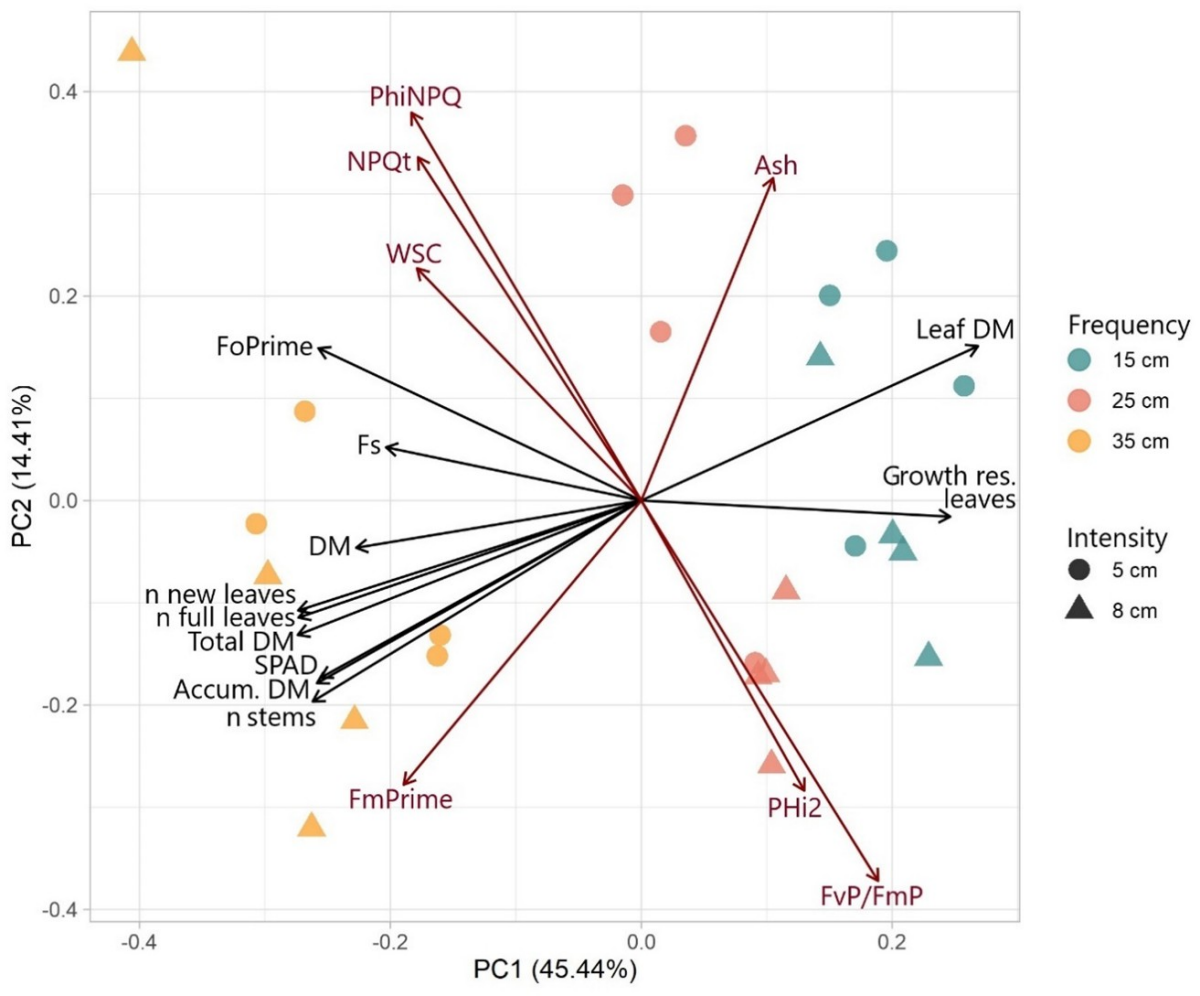
Biplot of PCA of plant characteristics. The principal components (PCs) were calculated with all measured variables of morphological and physiological traits, leaf and stem growth rate of leaves and stems, and nutritional composition. However, loadings are only presented for those variables with loadings greater than 0.20 or less than -0.20 for PC1 (black) or PC2 (dark red). F0Prime: minimum chlorophyll fluorescence (Fo´). FmPrime: maximum chlorophyll fluorescence (Fm´). Fs: steady‐state chlorophyll fluorescence. FvP/FmP: actual quantum efficiency of PSII (Fv′/Fm′). NPQt: total non-photochemical quenching. PHi2: quantum yield of PSII (Φ2). PhiNPQ: fraction of light dedicated to non-photochemical quenching (ΦNPQ). SPAD: relative chlorophyll content.

The PCA illustrates the relationships between morphological and physiological traits of plantain plants under different defoliation frequencies and intensities (Fig 2). The biplot of the PCA shows the variables with a loading greater than 0.20 or lower than -0.20 for PC1 or PC2. PC1 explained 45.4% of the variance and was strongly associated with above-ground productivity (number of new and fully developed leaves, number of stems, DM productivity, and growth of residual leaves). PC2 explains 14.4% of the variance and was strongly associated with physiological traits. Thus, PC1 could primarily be described as morphological traits of above-ground productivity. The vectors for total DM, number of new leaves, fully developed leaves, number of stems, and SPAD were closely aligned, indicating a positive correlation between these variables. Fo’ and Fs had a similar orientation on PC1, suggesting that these physiological traits are associated with above-ground productivity. Variables such as NPQt, ΦNPQ, WSC and ash loaded positively on PC2, and collectively reflect the relationship between these physiological traits and the accumulation of WSC, but they were inversely related to ϕ2 and Fv’/Fm’. The vectors for the number of new and fully developed leaves, number of stems and total DM loaded negatively on PC1, suggesting that these variables are inversely related to the growth of residual leaves and the accumulation of DM in these organs. Higher defoliation frequency (i.e. ELL of 15 cm) was associated with higher values on PC1, and samples subjected to a 25 cm defoliation frequency were clustered neutrally to positively along PC1. There was a complete separation between these groups and the lower defoliation frequency (ELL of 35 cm) group.

## Discussion

Before discussing our findings, we would like to address some potentially debatable aspects of the experimental design. A control treatment with undefoliated plants was not considered because the objective of the study was to evaluate different management strategies for defoliation of plantain pastures, as a feed source for ruminants. From that perspective, a treatment without defoliation would have no practical application for grazing production systems. Moreover, we followed a similar approach to that of Lee et al. [24] and Cranston et al. [10], where they tested different defoliation frequencies in plantain without undefoliated control. Studies conducted in controlled growth chambers have limitations in terms of space, resources, and management. Using 24 pots was a practical choice that maximized the use of available space without compromising the management and control of the experimental conditions such as light, temperature, and humidity. Having a manageable number of pots ensured that each plant received a uniform and consistent treatment. In greenhouse and growth chamber studies with forage species, pots with more than one plant are commonly used for several reasons, including: a) the simulation of plant interactions and natural competitions between plants for resources, b) increasing the statistical robustness of the results and enabling better assessment of variability among plants and environmental conditions, and c) optimizing space usage and increasing the amount of data collected in the experiment.

This study underscores the critical role of defoliation management, particularly in terms of frequency and, to a lesser extent, intensity, in optimizing regrowth capacity, forage production, and quality of *P*. *lanceolata* L. Additionally, it highlights the species’ physiological adaptability to varying defoliation conditions.

The first defoliation occurred when the plants averaged 24 cm of ELL and had at least six fully developed leaves as recommended by Powell et al. [14] for plantain pastures (cv. ’Ceres Tonic’). The reduced leaf length observed in our study, compared to the 30 cm reported by Powell et al. [14], could be attributed to decreased aboveground competition for resources such as light and nutrients under controlled environmental conditions and low plant density, resulting in reduced self-shading and, consequently, shorter leaves [44].

It is well-established that the ability of forage species to restore leaf area and resume photosynthetic activity depends on the frequency and intensity of defoliation [25, 26]. In this study, the regrowth dynamics and, consequently, herbage DM production in plantain plants were more influenced by defoliation frequency than residual height. The average total DM harvested per defoliation event increased by 185% and 278% per pot as the defoliation frequency increased from 15 to 25 cm and from 25 to 35 cm of ELL, respectively. Similarly, accumulated herbage DM production per pot increased during the 203 days of the experimental period as the defoliation frequency increased from 15 to 25 cm of ELL by 20.43 g per pot (which, under field conditions, equates to an increase in DM yield from 2.35 to 7.59 t DM/ha), but it was not affected by the 5 cm versus 8 cm defoliation intensity (residual height). Previous studies with plantain and chicory (*Cichorium intybus* L.), both of which have deeper root systems than PRG and WC (common forage species), conducted under field conditions, support the positive effect of decreasing defoliation frequency on herbage DM production [24, 29], while defoliation intensity showed no significant effect on herbage DM production [24]. Several studies have documented that an increase in the severity of defoliation (both in terms of frequency and intensity) reduces the regrowth capacity of forage species due to insufficient time to recover WSC reserves [25, 45–47]. After a defoliation event, the regrowth of the removed leaf tissue and resumption of photosynthetic activity depend on the plant’s short-term capacity to increase carbon dioxide (CO_2_) assimilation and chlorophyll content [25, 26], which, in turn, relies on the remaining photosynthetically active leaf area and the amount of carbon reserves primarily stored as WSC to support regrowth [26, 48]. In our study, more frequent defoliation at 15 cm of ELL likely affected the plantain’s capacity to support the regrowth of new leaf material forcing the mobilization of WSC reserves for regrowth [26, 49] and may not have been sufficient to achieve maximum DM accumulation. Based on these results, it could also be assumed that root development, and consequently the persistence of plantain pastures, would be affected by more frequent defoliation, as occurs in temperate grasses [26, 50, 51], especially under drier weather conditions. Defoliation intensity appeared to have little impact on the total WSC stored in the plant, probably because they are mainly stored in the roots (accounting for 70% of total WSC) rather than in the residual shoot tissue (30% of WSC) [52]. This contrasts with pastures based on the traditional PRG-WC or other forage species with shallow root systems, where an increase in the severity of defoliation (both frequency and intensity) reduces the capacity to restore leaf area and maintain herbage DM production because WSC reserves are mainly stored in the stubble [51].

In designing rotational grazing, the aim is to study the interaction between defoliation frequency and intensity on pasture regrowth capacity in order to determine the optimal grazing interval for forage species to recover [45, 53]. It was observed that defoliation frequency influenced plantain morphological traits. A higher defoliation frequency (i.e. ELL of 15 cm) led to an increase in the proportion of leaves in the total DM produced, consistent previous findings [24]. In contrast, less frequent defoliation resulted in longer leaves and stems, a greater proportion of reproductive stems in the total DM produced, and subsequently, higher DM production between cuts. The proportion of leaves in the total DM exceeded 80% in all treatments, surpassing values reported by Lee et al. [24] in field evaluations during the spring and summer seasons. This suggests that controlled growth conditions affect the proportion of leaves and stems in plantain.

The increase in reproductive components with low defoliation frequency alters the nutritional composition of plantain pastures, reducing digestibility, CP, and ash content [24]. In our study, conducted under controlled environmental conditions, all treatments exhibited high nutritive herbage quality. However, the increase in reproductive components, and consequently, the decrease in the leaf-to-stem ratio, in the forage cut at 35 cm of ELL increased the NDF proportion within total DM reducing the concentration of CP and ME and its nutritive value for livestock. Defoliation intensity also increased the proportion of stem in plantain with a residual height of 8 cm compared to 5 cm. These combined results underscore the importance of frequency and intensity in defining adequate defoliation management in plantain pastures. When defining optimal grazing management practices, it is desirable to maintain a greater proportion of leaves compared to reproductive stems to sustain high-quality pastures and enhance pasture use efficiency and animal yield. From a nutritional and productive perspective, defoliations with a frequency of ~15 days or 235 AGDD (25 cm of ELL) and residual heights close to 5 cm would allow for an increase in the quality of the forage produced without compromising the regrowth capacity of plantain. These findings are consistent with the results reported by Lee et al. [24] under mowing and Navarrete [32] under grazing conditions.

The practical context of this study pertains to defining the recommended frequency and intensity of defoliation in plantain to maximize DM production without compromising the nutritional quality of the forage. In the case of PRG, Fulkerson and Donaghy [26] proposed the "leaf stage grazing rule," suggesting that this species should be defoliated once it has reached the development of three leaves. This allows the plant to recover WSC reserves, facilitating adequate regrowth rates. For plantain, more frequent defoliations led to an increase in the phyllochron interval and a higher post-defoliation growth rate of the residual leaves in the 15 and 25 cm ELL treatments. Similarly, a higher growth rate of new leaves was observed in the less frequent defoliation treatments (ELL 25 and 35 cm), findings consistent with the work of Lee et al. [24]. Based on the results of this study, using less severe defoliation frequency seems to have a positive impact on DM production and regrowth rates in plantain. This suggests that plantain plants replenish their WSC reserves within the time required for the leaves to reach a length of 25 cm growing between 16°C and 26°C. Therefore, indicators based on leaf length could be useful for determining the best grazing practices for plantain. However, some flexibility should be considered when defining the optimum moment for defoliation based on leaf development indicators, as other factors, such as seasons, and the balance between quantity and quality of forage produced [54], may warrant adjustments.

Defoliation, often caused by livestock grazing, entails physical damage to plants, which can affect plant physiology, regrowth capacity, and persistence [55–57]. Chlorophyll fluorescence, generated during photosynthesis, provides insight into the physiological state of plants under both biotic and abiotic stress conditions [58, 59]. Previous studies have shown that plantain tolerates defoliation damage [56, 57]. However, basic fluorescence parameters (Fm’, Fo’, and Fs), as well as ΦNPQ and chlorophyll content, exhibited a plastic response to defoliation, increasing with decreasing cutting frequency. Minimum chlorophyll fluorescence (Fo) is often employed as an indicator of plant stress, as elevated Fo may signify damage to the photosynthetic apparatus [60], as observed in this case due to the least frequent cuts (35 cm). However, Fo’ and Fm’ displayed a similar trend, resulting in the absence of differences in the ratio of variable fluorescence to maximum fluorescence under light conditions (Fv’/Fm’), which serves as a measure of the actual efficiency with which plants convert light energy into chemical energy [61].

There were no differences in NPQ, which measures the amount of excess light dissipated as heat from PSII and thus indicates leaf photoprotection status [61], due to defoliation frequency or intensity. Consistent with this result, Barton et al. [62] also found no relationship between tolerance to herbivory leaf damage and NPQ levels after damage. However, NPQ has been reported to increase in plantain with defoliation [57]. On the other hand, ΦNPQ decreased with increasing cutting frequency. This decrease may indicate a loss of the leaf’s ability to dissipate excess energy as heat due to stress caused by more frequent defoliation and impairment of the photosynthetic apparatus. A similar trend was observed in the decrease in the SPAD index, which is also an indicator of increased stress with increasing defoliation frequency. Barton et al. [57] found a similar association with NPQ, rather than ΦNPQ, and chlorophyll content in plantain. This would suggest that increased defoliation frequency could restrict the ontogenetic development and complete maturation of the leaves. Therefore, although similar rates of chlorophyll biosynthesis might occur within the leaves across different treatments, chlorophyll accumulation in mature, non-senescent leaves was greater as defoliation frequency decreased. The increase in defoliation frequency restricted the ontogenetic display and complete maturation of the leaves. Hence, although similar rates of chlorophyll biosynthesis would occur within leaves of the different treatments, the accumulation of chlorophyll in mature, non-senescent leaves was higher as defoliation frequency decreased. Indeed, total chlorophyll content is higher in middle-aged or older leaves of species such as *Codiaeum variegatum* and *Fagus orientalis*, compared with younger leaves [63, 64].

Linear electron flow measures the electron transfer process within the photosynthetic electron transport chain [65]. This process is usually linearly correlated with net photosynthesis [66, 67]. Linear electron flow was the only chlorophyll fluorescence parameter that showed an interaction effect between defoliation frequency and defoliation intensity. There was no clear association between LEF and defoliation treatments, as LEF was highest in the most contrasting defoliation treatments, i.e., under the least intense and least frequent cutting and under the most intense and most frequent defoliation regime. The latter condition is considered highly stressful. The high LEF in the most stressful conditions could be related to increased photorespiration or the water-water cycle, mechanisms that help the plant withstand the stress of cutting [68]. Both processes contribute to maintaining a high linear electron transport and the transthylakoid pH gradient to deal with oxidative stress [68–71]. Thus, the increase in electron transport chain activity through PSII suggests that plants are employing mechanisms to maintain optimal photosynthetic activity and cope with stressors, such as an increase in reactive oxygen species (ROS) [72]. However, in this case, the elevated LEF may not effectively contribute to productive photosynthesis and may affect growth and productivity. On the other hand, high LEF in the less intensive and less frequent treatment may be associated with better photosynthetic performance, consistent with a high proportion of open reaction centers (qL) and a concomitantly better Φ2 and a higher amount of total carbohydrates. This could be related to plantain achieving expanded leaves with "mature" photosystems and experiencing lower oxidative pressure due to less frequent and less intensive cutting. Therefore, the intensity and frequency of defoliation affect photosynthetic performance, but the specific processes affected depend on whether the plant must deal with oxidative stress or maintain carbon gain. Overall, plantain displayed resilience to defoliation management as indicated by the chlorophyll fluorescence parameters, such as Φ2, and the resulting lack of differences in the actual quantum efficiency.

This study suggests that plantains have the potential to tolerate more frequent defoliation intervals, ranging from 9 to 16 days or 142 to 235 AGDD at 15 and 25 cm ELL, respectively. This tolerance is achieved through increased energy allocation towards reestablishing foliar area. This strategy promotes greater growth of residual leaves and enhances the Fv’/Fm’ ratio and Φ2, increasing the proportion of leaves in the total DM. On the other hand, less frequent defoliation led to changes in morphological composition, including an increase in the proportion of reproductive stems, which subsequently reduced herbage nutritive value for ruminants. However, it is important to note that under real grazing conditions, repeated treading damage caused by high grazing frequency could potentially reduce plantain content and persistence in the pasture [73]. This effect may be exacerbated if other abiotic stresses, such as water restrictions, are also present.

## Conclusions

The study of the defoliation dynamics of *P*. *lanceolata* L. under controlled conditions provides valuable insights into the effects of defoliation on herbage production, quality, morphological composition and photosynthesis. Less frequent defoliation led to increased leaf growth and length, as well as higher herbage dry matter production. Maintaining a 15-day defoliation frequency at 25 cm of extended leaf length promotes better regrowth and forage yields, while maintaining a greater proportion of leaves ensures high nutritional value, demonstrating a favorable balance between quality and regrowth capacity in *P*. *lanceolata* L. Chlorophyll fluorescence analysis revealed intricate responses to defoliation frequency, indicating the plant’s ability to acclimate and exhibit resilience to cutting stress. This study contributes to the understanding of optimal grazing practices for *P*. *lanceolata* L., promoting higher herbage production and improved forage quality while considering the plant’s responses to defoliation. These findings hold practical significance for sustainable pasture management.

## Supporting information

S1 Dataset (XLSX)
